# The Elephant in The Room: AML Relapse Post Allogeneic Hematopoietic Cell Transplantation

**DOI:** 10.3389/fonc.2021.793274

**Published:** 2022-01-03

**Authors:** Iman Abou Dalle, Ali Atoui, Ali Bazarbachi

**Affiliations:** Department of Internal Medicine, American University of Beirut Medical Center, Beirut, Lebanon

**Keywords:** AML, allotransplant, relapse, immunotherapy, Graft versus leukaemia (GVL)

## Abstract

Relapsed acute myeloid leukemia (AML) following allogeneic hematopoietic cell transplantation (allo-HCT) is an unfavorable event associated with a poor prognosis, particularly for patients with early relapses. It usually arises from resistant leukemic blasts that escaped both preparative chemotherapy regimen and the graft-versus-leukemia (GVL) effect. Independent from the choice of salvage treatment, only minority of patients can achieve durable remissions. In recent years, better understanding of the disease relapse biology post allo-HCT allowed the application of newer strategies that could induce higher rates of remission, and potential longer survival. Those strategies aim at optimizing drugs that have a direct anti-leukemia activity by targeting different oncogenic mutations, metabolism pathways or surface antigens, and concurrently enhancing the immune microenvironment to promote GVL effect. This review discusses the current treatment landscape of AML relapse post allo-HCT.

## Introduction

Allogeneic hematopoietic cell transplantation (allo-HCT) continues to serve as a potentially curative step in the management of intermediate- and high-risk acute myeloid leukemia (AML) (1). Nowadays, virtually all patients can benefit from allo-HCT owing to the advances in the field from the use of reduced-intensity conditioning (RIC) for older and/or unfit patients, to the availability of alternative donors, and the improvement in supportive care and graft-versus-host disease (GVHD) prophylaxis. Over the past 40 years, the non-relapse mortality (NRM) at one year has declined significantly from 24% in the 1990s to less than 10% from 2013 through 2016 (2). However, disease relapse remains the main treatment failure following allo-HCT, and is associated with dismal outcomes and a median overall survival (OS) of few months (3–5). The cumulative incidence of relapse is largely dependent on the disease risk, the intensity of conditioning regimen, and the type of donor used, varying between 15-20% after a myeloablative conditioning, 30-50% after RIC regimen, and up to 58% when RIC is used for haploidentical transplants (6–9). Majority of the relapses occur within the first year after allo-HCT.

AML relapse post allo-HCT poses a serious challenge to clinicians, as despite increasing survival rates of younger patients relapsing post allo-HCT, their 2-year OS didn’t exceed 26% (3, 10, 11). A minority of patients can be rescued with a second allo-HCT which also provides a long-term survival to no more than one-third of the patients (10). In front of an elephant in the room, traditional therapies have proven to be ineffective in attaining long-term remissions without toxicity and newer therapeutic approaches have become a necessity.

**Figure 1:**
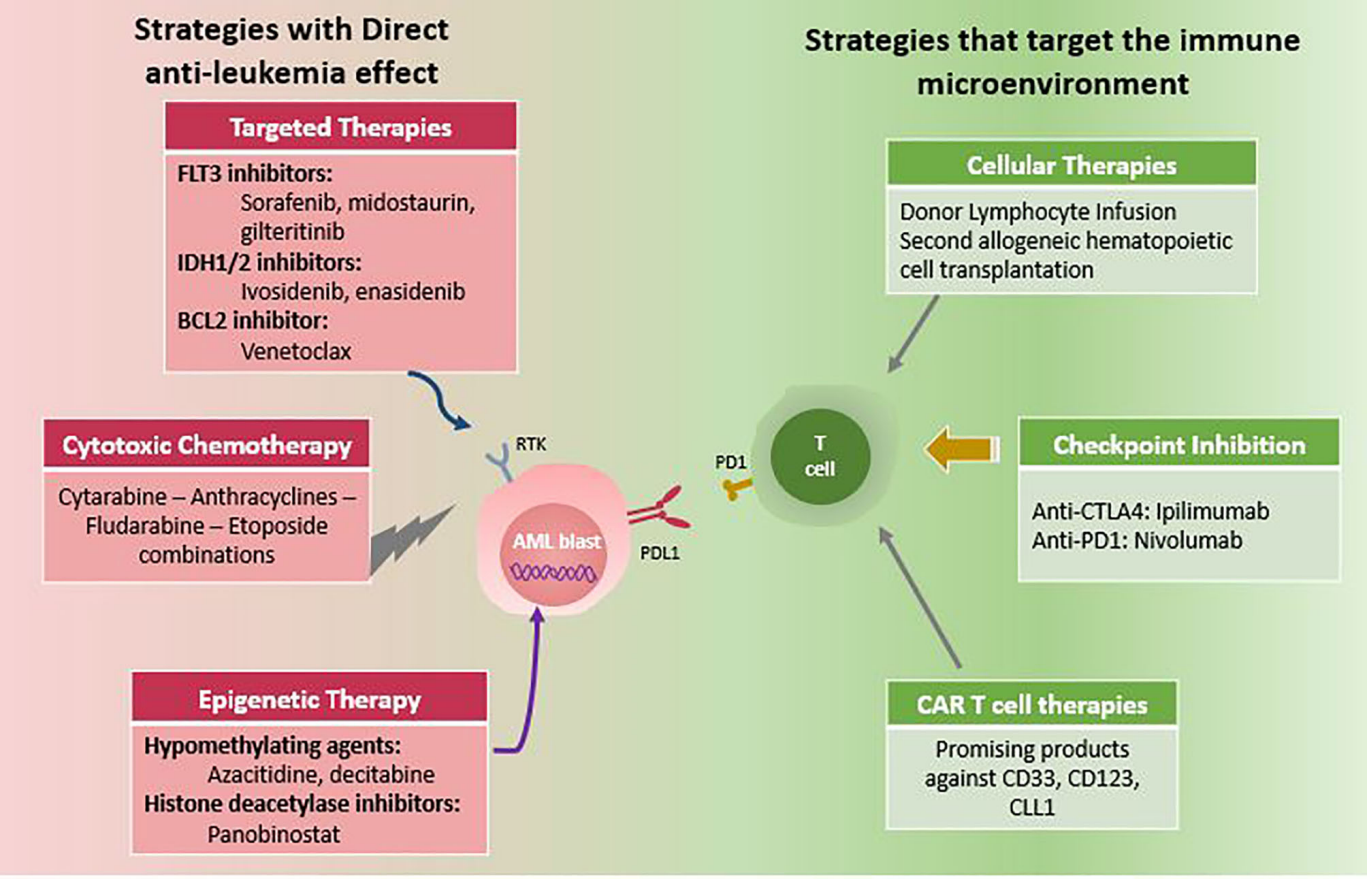
Therapeutic options for post-transplant AML relapse. Note: Combination of two or more options are increasingly used.

Understanding the factors that led to disease relapse will help in developing and exploring rationally designed combination therapies. First, relapsing leukemia cells showed resistance to preparative chemotherapy either during induction/consolidation or within the conditioning regimen, but most importantly, they had definitely an immune escape mechanism to evade the donor-derived T cells. With the advent of new sequencing technologies, many oncogenic targets have been discovered and served as an additional tool in the armamentarium of AML management. The main goal of treatment is first to eradicate leukemia cells using effective anti-leukemia therapies, and then activate the donor alloreactive T cells toward a graft-versus-leukemia (GVL) direction. This review will discuss current therapeutic options including those directed against leukemia cells, in addition to other strategies aiming at enhancing the GVL effect of immune cells (**Figure 1**).

## Biology of Relapse Post Allo-HCT

Discussing the different escape mechanisms by which leukemia usually relapse post allo-HCT is not within the scope of this review, however our understanding of the biology of disease relapse will help develop strategies to prevent and/or treat AML in the post allo-HCT setting. Multiple patient-, disease-, and transplant related factors are implicated in an increased risk of relapse post allo-HCT, including older age, high-risk cytogenetics, the presence of *FLT3* mutation, the absence of *NPM1* mutation, the time from achieving remission to allo-HCT, the absence of GVHD and the *in-vivo* T-cell depletion (12). These factors contribute differently in early and late relapses (12). Early relapses are usually driven by a highly proliferative disease that outstrip the rate of GVL development. The genetic biology of AML plays an important role in determining subgroups of patients with chemoresistance (13). For example, *FLT3*-ITD mutation is known to be associated with a high risk of relapse and worse survival post allo-HCT (14). Mutations in *WT1* and *TP53* mutations are also associated with higher risk of relapse post allo-HCT and worse survival (15). Moreover, AML consists usually of multiple genetically distinct subclones that are variable in their phenotype and frequency. These subclones contain different numbers of leukemia initiating cells with self-renewal potential (16). The anti-leukemia therapy exerts different pressures on these subclones, leading to clonal evolution and emergence of therapy-resistant population that cause AML relapse.

In the post allo-HCT setting, the mechanism of disease relapse becomes more complex, involving the immune microenvironment. Multiple mechanisms have been described including the dysregulation in pathways involved in adaptive and innate immunity, such as the downregulation of major histocompatibility complexes (MHC) Class II genes (HLA-DPA1, HLA-DPB1, HLA-DQB1, and HLA-DRB1), the downregulation of NK cell targets, the loss of expression of an HLA haplotype, and the increased expression of inhibitory checkpoint ligands (17–20).

## Strategies With Direct Anti-Leukemia Effects

The initial goal in treating AML relapse post allo-HCT is to decrease the leukemia burden and ultimately achieve a deep complete remission (CR) that is sustainable. Among therapies with direct anti-leukemia effect, is the use of intensive cytotoxic chemotherapy. Despite the rapid debulking effect, a minority of patients can tolerate such treatment especially in the early post-transplant phase. In addition, the use of chemotherapy alone is generally not sufficient with a 2-year OS not exceeding 7% (7). The CR rates post intensive chemotherapy rarely exceed 30% to 40% with an increased risk of treatment-related mortality to 20%. Patients with a post-transplant remission duration longer than 6 months seem to better tolerate the treatment, and have a higher likelihood of response. Those who successfully respond to intensive chemotherapy, and were subsequently bridged to a second allo-HCT will have a 2-year OS of 40% (21, 22).

The best results to date have been achieved after donor cell therapies either with donor lymphocyte infusion (DLI) or a second allo-HCT with 2-year OS rate between 20 and 40% (21, 23). Better outcomes are usually encountered in patients who achieved morphologic CR or low burden of disease at time of cellular therapy (3). Thus, other effective anti-leukemia strategies capable of achieving remissions are extremely needed. These can involve targeting tumor-specific surface antigens, cellular metabolism, and activating oncogenic mutations. 

### Hypomethylating Agents (HMAs)

Hypomethylating agents (HMAs) such as azacitidine and decitabine are effective anti-leukemia drugs with a proven efficacy in the frontline treatment of AML in older patients. Their use as single agents resulted in improvements in clinical outcomes of older AML patients with a median OS of 8-10 months (24, 25). Due to its efficacy and tolerability, HMA therapy was largely used by many clinicians in the treatment of AML relapse post allo-HCT (26, 27). Initially, it was thought that HMAs are capable of activating silenced tumor suppressor genes through hypomethylation, however many other mechanistic actions were discovered throughout the years, contributing to their effectiveness in the post-transplant setting. By increasing the expression of epigenetically silent tumor antigens, azacitidine can enhance the GVL effect by activating CD8+ T cells directed toward many tumor antigens like melanoma-associated Ag 1, B melanoma antigen 1, and Wilm tumor Ag 1 (28). Concurrently, azacitidine can expand regulatory T cells, thus decreasing the risk of GVHD (29).

Despite the excitement regarding the use of HMAs in the post-transplant relapse, limited number of patients responded, and long-term survival was not possible. Craddock et al. reported the outcomes of patients with AML or high-risk myelodysplastic syndrome (MDS) treated with azacitidine +/- DLI for post-transplant relapse (27). The CR rate was only 15% after a median of 3.6 months. The 2-year OS for all patients was 12.4%, but 48% for those who responded to azacitidine. Interestingly, the addition of DLI to the regimen did not impact the response rate nor the survival (27). Many other retrospective trials investigated the use of DLI in addition to azacitidine or decitabine and resulted in an overall response rate (ORR) of 25-33%, and a 2-year OS between 11% and 29% (30–32).

With the purpose of enhancing the effect of azacitidine, multiple combination therapies were assessed. The combination of azacitidine with lenalidomide might have an additive anti-leukemic effect, while azacitidine decreases the risk of acute GVHD from lenalidomide (33). In the VIOLA trial, 29 patients were treated with standard dose azacitidine (75 mg/m2 for 7 days) with escalated doses of lenalidomide (5-25 mg daily), 15 patients received at least 3 cycles of treatment with modest toxicity, and no increased rates of GVHD. Among evaluable patients, the ORR and CR rates were 47% and 20% respectively, considered to be slightly higher than azacitidine alone when compared to previous reports. Those who responded had a better median OS of 27 months compared to 10 months in non-responders (p=0.004) (33).

The treatment of AML with novel agents is fast evolving, with multiple targeted agents already FDA approved in different settings. Currently, many new targeted agents are effective in AML, and can be used in the post-transplant relapse setting (34). HMAs can also be the backbone treatment for any combination treatment with targeted agents such as BCL2-inhibitors, FLT3 inhibitors and IDH1/2 inhibitors.

### BCL2 Inhibitors

Venetoclax is an orally available selective BCL2 inhibitor, that competitively combines to BH3 domain and induces apoptosis of leukemia stem cells. It is currently approved by the Food and Drug Administration (FDA) in combination with azacitidine or low dose cytarabine in newly diagnosed AML patients older than 75 years or unfit for intensive chemotherapy (35, 36). HMA and venetoclax have significantly improved the outcomes of newly diagnosed AML patients with a reported median OS of 14 months and an acceptable toxicity profile (35). The combination regimen has been also used in the relapsed and/or refractory (R/R) setting with an ORR of 21% and short survival (37). In the post-transplant relapse setting, azacitidine and venetoclax were used in a limited number of patients, with a reported ORR of 38-46% (38, 39). More recently, a prospective clinical trial combining FLAG-Ida regimen with venetoclax in the R/R setting reported a composite CR rate of 57% among patients with prior allo-HCT, with a 75% rate of second allo-HCT among responders (40).

Further ways to overcome venetoclax resistance and enhance its efficacy is by targeting MCL1, an anti-apoptotic protein. A recently reported study combined actinomycin D, with azacitidine and venetoclax for the treatment of AML relapse post allo-HCT. The rationale of adding actinomycin D was based on preclinical data suggesting a synergistic anti-leukemic effect through MCL1 degradation, in addition to mitochondrial activity yielding to senescence through PML-nuclear body biogenesis (41–43). Twenty patients were treated with the triplet combination with an impressive ORR of 75% and a median OS of 13.1 months (41). venetoclax- combination therapies have become a new standard of care even in the post-transplant setting.

### IDH1/2 Inhibitors

Somatic mutations in isocitrate dehydrogenases (IDH) 1 and 2 occur in up to 20% of AML patients (44). Targeted agents have been approved in the treatment of R/R AML harboring *IDH1* or *IDH2* mutations. Enasidenib monotherapy yielded an ORR of 40% with a median OS of 9.3 months, reaching 19.7 months in patients attaining CR in the R/R setting (45, 46). In this trial, 36 patients with *IDH*2 mutant AML had prior allo-HCT when treated with enasidenib (45). Similarly, 258 patients with R/R *IDH1* mutant AML including 79 patients receiving prior allo-HCT were treated with ivosidenib monotherapy (47). The reported ORR was 41.9% with durable responses. Both drugs are very well tolerated. Side effects of special interest include indirect hyperbilirubinemia and IDH-inhibitor related differentiation syndrome. In the setting of R/R AML post allo-HCT, both enasidenib and ivosidenib can be used in combination with HMAs and venetoclax.

### FLT3 Inhibitors

Patients with AML who relapse post allo-HCT may harbor a *FLT3* internal tandem duplication (ITD) mutation that is usually associated with poor outcomes. Sorafenib is a first generation tyrosine kinase inhibitor that targets *FLT3*-ITD mutation. In addition to its anti-FLT3 activity, sorafenib has been shown to induce interleukin-15 production by FLT3-positive leukemia cells, thereby activating the donor CD8+ T cells and promoting the GVL effect (48). In the post-transplant relapse setting, sorafenib as monotherapy resulted in durable remissions indicating a clinical synergy with the allogeneic immune effects (49, 50). In a retrospective analysis from the European Bone Marrow Transplantation (EBMT) registry, 30 patients were treated with sorafenib as single agent for relapse post allo-HCT; the CR rate was 39% among evaluable patients. When compared to matched controls, the 2-year OS was 38% compared to 9% for controls (p=0.0001) (51). Gilteritinib is a second generation, more potent FLT3 inhibitor that targets both *FLT3*-ITD and *FLT3*-TKD mutations. In the ADMIRAL phase 3 trial, 371 patients with R/R *FLT3* mutant AML were randomly assigned to receive either gilteritinib at 120 mg daily or salvage chemotherapy (52). Gilteritinib resulted in a higher CR/CR with incomplete hematologic response rate (34% versus 15%, respectively), and a longer median OS (9.3 months versus 5.6 months, p<0.001). In the trial, 20% of patients were relapsing post allo-HCT. Among those patients, the CR rate achieved with gilteritinib was 35.4% versus 11.5% for those who received salvage chemotherapy. The median OS was also prolonged with gilteritinib compared to chemotherapy (8.3 months versus 4 months, Hazard Ratio: 0.48 (95% Confidence Interval:0.27-0.84) (52).

### Innovative Therapies

Although this review focuses on the current treatment landscape of AML relapse post allo-HCT, it is worth noting that several novel therapeutic options are ongoing investigation. Many of these agents are already FDA approved, such as glasdegib, an oral small molecule inhibitor of the Smoothened (SMO) receptor, inhibiting the Hedgedog signaling pathway. Glasdegib was tested in combination with low dose cytarabine in the frontline treatment of AML in patients who are ineligibile for intensive chemotherapy (53). It was used in the R/R setting with modest efficacy (54). Other novel agents include MCL1 inhibitors, MDM2 inhibitors especially in combination with venetoclax (55, 56).

## Strategies That Target the Immune Microenvironment

### Donor Lymphocyte Infusion (DLI)

DLI is an adoptive immunotherapy treatment defined as transfusion of non-stimulated lymphocyte concentrate from the original stem cell donor. It was first introduced in the early 1990s and showed high efficacy in patients with relapsed chronic myeloid leukemia after allo-HCT (57, 58). The understanding of the potent GVL effect of DLI has led to its widespread adoption in the management of relapsed hematological malignancies after allo-HCT. However, complications such as acute GVHD remain the main concern. In a retrospective study evaluating the role of DLI in the treatment of relapsed AML after allo-HCT from the EBMT, the estimated survival at 2 years was significantly higher in patients receiving DLI compared to those not receiving DLI (21% vs 9%, respectively). Lower tumor burden, favorable cytogenetics and remission at time of DLI were associated with better survival in DLI recipients. The overall incidence of acute GVHD was 43% at day 100 after DLI (59). This limited efficacy of DLI in the treatment of AML relapse after allo-SCT was confirmed by another retrospective data from the AML working group of the Japan society of hematopoietic cell transplantation. Out of 143 patients treated with DLI at first relapse, OS rates were 32% at 1 year, 17% at 2 years and 7% at 5 years. Long-term survival was almost exclusively associated with CR before DLI (2-year OS of 100%), confirming the necessity of achieving a clinical response before DLI. Acute GHVD was reported in 18% of patients (60). The efficacy of therapeutic DLI was shown to be inferior in the context of hematological relapse compared to mixed-chimerism. In a retrospective study from the Italian group including 180 patients with relapsed AML, the 3-year OS was significantly higher in patients who has received DLI for mixed chimerism compared to those with acute leukemia relapse (55% vs 32% respectively, p=0.002). Although published studies have suggested that outcomes of haplo-identical and HLA-matched DLI are comparable in terms of outcomes with similar incidence of DLI-induced acute GVHD, it is suggested that haplo-DLI might be more beneficial in promoting GVL effect (61).

### Second Allogeneic Stem Cell Transplantation

Patients with relapsed AML after allo-HCT who are eligible for intensive treatment are usually offered a second allo-HCT. However, there is currently no standard of care regarding the donor selection and the type of conditioning regimen used in this setting. In a registry-based study of 418 patients with AML comparing survival at first relapse after allo-HCT, there was no significant difference in OS between the two groups regardless of the disease status at time of treatment. The 2- year and 5-year OS was 25% and 15% respectively in the DLI group compared to 26% and 19% in the allo-HCT group. However, the incidence of NRM was significantly higher in patients who received a second allo-HCT compared to those who received DLI including all patients and those who received treatment with active disease (2-year NRM 9% compared to 26% and 5-year NRM 10% compared to 29% in the DLI group compared to allo-HCT group, respectively). NRM was not significantly different in patients who were in CR at the time of intervention. The rate of grade 2 to 4 acute GVHD was significantly higher in the allo-HCT group (37%) compared to DLI group (20%). Interestingly, there was no difference in the 2-year OS and NRM in patients who received a second allo-HCT by donor type (62). The impact of donor selection on outcome of second allo-HCT was addressed by a study from the Acute Leukemia Working Party of EBMT. The retrospective analysis included 556 patients divided into 3 groups based on allo-HCT donor: same donor, different matched related, and haplo-donor. The 2-year leukemia-free survival (LFS) was comparable between the three groups (LFS 23% for same donor, 23.7% for different matched donor and 21.8% for haplo-donor, p=0.3). Similarly, there was no difference in the 2-year OS between the groups (36.4%, 28.7% and 23.3%, respectively. p=0.21). However, the cumulative incidence of grade 2 to 4 acute GVHD was significantly higher in the same donor group (63). Although it is thought that a haplo-identical donor after a matched related donor might offer an advantage in terms of GVL effect, the outcome of a second allo-HCT is equivalent and the use of a haploidentical donor is not usually associated with better survival. In the setting of allo-HCT for relapsed AML post allo-HSCT, the choice of the donor is mainly limited by the availability as there is no current data to support donor selection based on outcomes and complications of transplant.

### Checkpoint Inhibitors

Other immune strategies to enhance the T cell activity in AML, especially in the post-transplant setting involve the use of immune checkpoint inhibitors. Although these compounds are not yet approved in the management of AML, however they are gaining more clinical interest especially after a phase 1/1b clinical trial reporting a promising anti-leukemia efficacy of high dose ipilimumab, an anti-CTLA4 antibody, in a subset of AML patients relapsing post allo-HCT (64). In fact, it has been proven that AML cells are capable of evading T-cell responses through the overexpression of PD-L1, the ligand of PD-1 (65). This overexpression was significantly higher in patients with relapsed AML than in untreated patients (65). Furthermore, it appears that HMAs can induce an upregulation of the inhibitory immune checkpoint molecule expression as part of resistance mechanism (66). Thus the rationale for combining azacitidine with nivolumab in relapsed and/or refractory AML (67). In a phase 2 trial, 70 patients with R/R AML were treated, and achieved an ORR of 33% with an encouraging median OS of 10.5 months in patients with first salvage (67). Despite the possibility of durable responses with immune checkpoint inhibitors post allo-HCT relapse, immune mediated adverse effects and exacerbation of severe fatal GVHD remain the main concerns of such strategy.

### Other Cellular Therapies

The field of cellular therapy is fast evolving in the management of hematologic malignancies, especially with the approval of CAR T cells for B-cell acute lymphoblastic lymphoma and non-Hodgkin’s lymphoma. In AML, the development of CAR T cells remain problematic, mainly because many myeloid surface antigens on AML cells, are also expressed on normal hematopoietic or progenitors cells, causing severe bone marrow suppression. Currently, many CAR T cells products are being investigated in AML targeting CD33, CD123, and CLL1 that are predominately expressed on AML blasts (68, 69).

## Conclusion

Despite all advances in the field of allo-HCT in AML, disease relapse still represents a major challenge with very limited treatment options. Many factors contribute to disease relapse including clonal evolution with acquisition of new oncogenic mutations, immune escape mechanisms such as downregulation of MHC genes, loss of expression of HLA haplotype, downregulation of NK cells targets, and increased expression of immune inhibitory checkpoint ligands. This progress in our understanding of the biology of disease relapse post allo-HCT is being translated in new therapeutic strategies. Epigenetic regulation using HMAs showed to be effective either alone or with DLI. Immune checkpoint blockade by ipilimumab was also feasible post allo-HCT relapse, particularly in patients with extramedullary involvement (64). More targeted agents are also promising in the post-transplant relapse setting, including FLT3 inhibitors, IDH1 and IDH2 inhibitors, as well as the BCL2 inhibitor, venetoclax. Definitely, there is still potential for improvements in the choice of treatment in the post-transplant relapse setting toward a more personalized approach. Pre-emptive and maintenance strategies post allo-HCT to prevent the relapse to occur is also imperative (70). Enhancing the GVL effect using HMAs is a promising approach, as well as the use of maintenance tyrosine kinase inhibitors such as FLT3 inhibitors post allo-HCT (71–76). Moreover, progress in accurate quantification and monitoring of measurable residual disease post allo-HCT is helpful in predicting impending morphologic relapse that prompts treatment initiation (77, 78).

## Author Contributions

IA, AA, and AB reviewed the literature and wrote the manuscript. All authors contributed to the article and approved the submitted version.

## Conflict of Interest

The authors declare that the research was conducted in the absence of any commercial or financial relationships that could be construed as a potential conflict of interest.

## Publisher’s Note

All claims expressed in this article are solely those of the authors and do not necessarily represent those of their affiliated organizations, or those of the publisher, the editors and the reviewers. Any product that may be evaluated in this article, or claim that may be made by its manufacturer, is not guaranteed or endorsed by the publisher.
